# Advances in Polysaccharide- and Synthetic Polymer-Based Vitreous Substitutes

**DOI:** 10.3390/pharmaceutics15020566

**Published:** 2023-02-08

**Authors:** Kruti Naik, Lisa C. Du Toit, Naseer Ally, Yahya E. Choonara

**Affiliations:** 1 Wits Advanced Drug Delivery Platform Research Unit, Department of Pharmacy and Pharmacology, School of Therapeutic Sciences, Faculty of Health Sciences, University of the Witwatersrand, 7 York Road, Parktown, Johannesburg 2193, South Africa; 2 Division of Ophthalmology, Department of Neurosciences, School of Clinical Medicine, Faculty of Health Sciences, University of the Witwatersrand, 7 York Road, Parktown, Johannesburg 2193, South Africa

**Keywords:** retinal detachment, vitrectomy, vitreous substitute, in situ hydrogel, polysaccharide, synthetic polymers, artificial vitreous

## Abstract

The vitreous humour is a gel-like structure that composes the majority of each eye. It functions to provide passage of light, be a viscoelastic dampener, and hold the retina in place. Vitreous liquefaction causes retinal detachment and retinal tears requiring pars plana vitrectomy for vitreous substitution. An ideal vitreous substitute should display similar mechanical, chemical, and rheological properties to the natural vitreous. Currently used vitreous substitutes such as silicone oil, perfluorocarbon liquids, and gases cannot be used long-term due to adverse effects such as poor retention time, cytotoxicity, and cataract formation. Long-term, experimental vitreous substitutes composed of natural, modified and synthetic polymers are currently being studied. This review discusses current long- and short-term vitreous substitutes and the disadvantages of these that have highlighted the need for an ideal vitreous substitute. The review subsequently focuses specifically on currently used polysaccharide- and synthetic polymer-based vitreous substitutes, which may be modified or functionalised, or employed as the derivative, and discusses experimental vitreous substitutes in these classes. The advantages and challenges associated with the use of polymeric substitutes are discussed. Innovative approaches to vitreous substitution, namely a novel foldable capsular vitreous body, are presented, as well as future perspectives related to the advancement of this field.

## 1. Introduction

**Figure 1:**
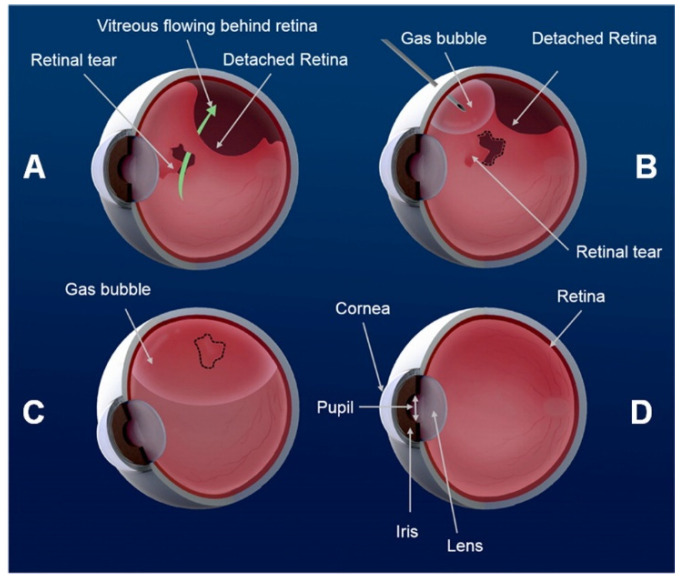
Endotamponade caused by an intraocular gas bubble during pneumatic retinopexy. (**A**) Fluid enters behind the retina causing detachment. (**B**,**C**) Retinal reattachment is facilitated through gas bubble tamponade. (**D**) Reattachment of the retina. Reprinted with permission from [11] Copyright 2015 American Chemical Society.

The World Health Organisation (WHO) estimates that 2.2 billion people suffer from vision impairment or blindness globally. Of these 2.2 billion, 1 billion people have preventable causes of blindness or visual impairment [1]. Blindness and visual impairment can be caused by, cataracts, glaucoma, diabetic retinopathy, age-related macular degeneration (AMD), and retinal detachment (RD) [1,2]. The leading cause of global blindness is cataracts, while glaucoma is the leading cause of irreversible visual loss and is second to cataracts as a cause of blindness globally [3,4]. Worldwide, an estimated 14 million people are affected by age-related macular degeneration [5] and one-third of people suffering from diabetes mellitus (285 million) are affected by diabetic retinopathy [6]. Pars plana vitrectomy (PPV) is one of the most commonly used treatments for many retinal diseases, especially RD [7]. During PPV the vitreous body is removed and replaced with a vitreous substitute [8]. Certain types of RD can also be treated with pneumatic retinopexy (PR) [9]. PR is an outpatient procedure that involves the induction of retinopexy around the retinal breaks or tears and intravitreal injection of a long-acting expansive gas (Figure 1) [10].

**Figure 2:**
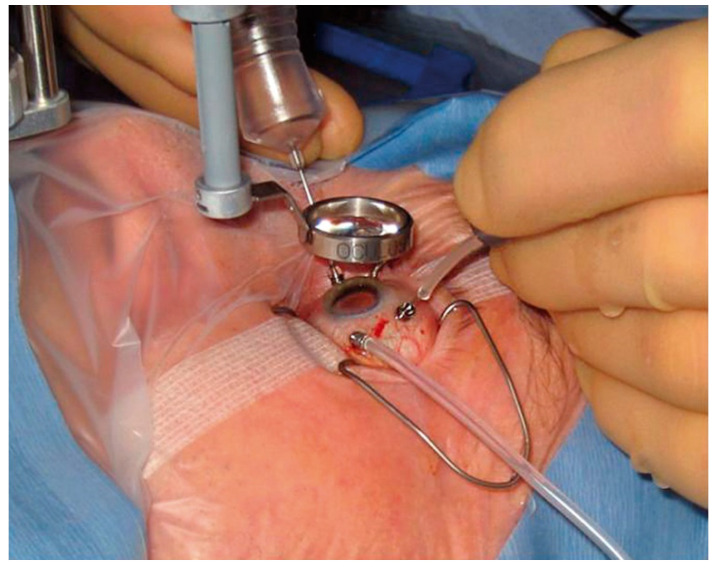
During vitrectomy surgery, microsurgical instruments are placed in the eye through incisions in the sclera. A suitable substitute is used to replace the native vitreous [13].

The purpose of the vitreous substitute is to fill the vitreous cavity and to help reattach the vitreous and the retina after vitrectomy surgery (Figure 2) by providing tension on the eye and promoting healing [8,12]. Silicone oil and gases are commonly used for medium and long-term tamponade effects, while perfluorocarbons are utilised as intraoperative tamponades. Currently, the only post-operative choice for long-term vitreous substitution is silicone oil [7,12]. The optical clarity and chemical inertness of these compounds are favourable, but many disadvantages and adverse effects have prompted researchers to develop a vitreous substitute using polymers.

The development of an ideal vitreous substitute is one of the most intricate and difficult disciplines of ophthalmic research and a suitable polymeric substitute is yet to be developed. This review presents and critically evaluates current vitreous substitutes with emphasis placed on developments in polysaccharide and synthetic polymer hydrogels that show promise. For this review, a literature search was conducted for the keywords “vitreous substitute”, “artificial vitreous”, “experimental vitreous”, “polymeric vitreous”, “vitreous mimetic”, “vitreous humour”, “retinal tamponade”, “retinal detachment”, “silicone oil substitute”, and “hydrogel vitreous”. Databases utilised for the search include SCOPUS, Google Scholar, and PubMed. Papers from 1950 were included in the search and references in the relevant articles were also used. Articles in languages other than English were excluded.

## 2. The Natural Vitreous

The vitreous is a transparent, gel-like structure, with a volume of approximately 4 millilitres, occupying the majority of each eye [14]. The vitreous provides a clear passage of light, provides oxygen transport through the eye, and also functions to hold the retina in place, dampen eye movements, and create a barrier against biochemical substances [15,16,17]. The gelatinous structure of the vitreous is based on the polysaccharide, hyaluronic acid, and the peptide, collagen, which, along with 98% water, are the main components of the vitreous [15,18]. Structural stability of the vitreous is provided through the binding of water to proteins and glycosaminoglycans (GAGs) [17]. The proteins, GAGs, metabolites, and cells found in the vitreous are shown in Table 1 [17,18].

The vitreous has a density of 1.0053–1.008 g/cm^3^, a refractive index of 1.3345–1.3348, and a pH of 7.0–7.4 [18]. A high hyaluronic acid concentration with suspended collagen fibres within the vitreous allows for the viscosity of the vitreous (300–2000 cP) and absorbs the stress and strain created during the movement of the eye during the day. The viscosity of the vitreous is highest close to the posterior pole of the eye and decreases as you move anteriorly [15]. Collagen, GAGs, and water in the vitreous ensure transparency and support the mechanism responsible for vision and accommodation of the eye [15,18]. Ascorbic acid in the vitreous has been found to act as a neovascularisation inhibitor; it can increase the proliferation of hyalocytes, and act as an antioxidant to reduce free oxygen in the vitreous [12,18].

The vitreous humour is a viscoelastic gel or solid displaying both solid- and liquid-like behaviour. The storage shear modulus (G’) of the vitreous is higher than the loss shear modulus (G”). The storage modulus represents the recoverable or elastic component while the loss modulus represents the viscous component [19,20]. Therefore, under static loading, the vitreous will respond as an elastic body but during rapid unloading events, such as trauma, the vitreous will be capable of dissipating substantial energy [15].

## 3. The Ideal Vitreous Substitute

The ideal vitreous substitute should have analogous viscoelastic properties to the natural vitreous. It should maintain intraocular pressure within a normal range, and it should be able to support the surrounding ocular tissue such as the retina without causing toxicity [12]. Vitreous substitutes must be transparent and permeable. The natural vitreous maintains the shape of the eye by behaving as a viscoelastic fluid or gel [12,21] and a vitreous substitute must mimic this. An ideal vitreous substitute should also not degrade with time [15].

## 4. Current Vitreous Substitutes

During pars plana vitrectomy, the vitreous is removed to repair a retinal detachment or clear a vitreous haemorrhage. Silicone oils, perfluorocarbon liquids, gases, air, and balanced salt solutions are substitutes that are currently used following vitrectomy [12,22]. These substitutes work as short-term or long-term tamponades that keep the retina in place. Table 2 shows various gas and liquid tamponade agents used. Polymeric vitreous substitutes are currently only used for sustained drug delivery mainly for posterior segment diseases and have not yet been employed as permanent vitreous substitutes [23].

### 4.1. Gases

Vitreous substitutes with gases are used for pneumatic retinopexy and post-operative endotamponade. These include the use of air and expansile gases such as sulfur hexafluoride (SF_6_), perfluoroethane (C_2_F_6_), and perfluoropropane (C_3_F_8_) [12,24,25].

#### 4.1.1. Air

Treatment of retinal detachment (RD) was first conducted by Ohm in 1911 using sterile air in an intravitreal injection [7]. Air is readily available, inexpensive, and does not need to be removed as it is replaced with aqueous humour once absorbed [18]. However, the refractive index of air is incompatible with ocular tissue, and it has a decreased residence time [24]. Thus, air is only used when no other substitutes are available [12], but recent studies show that air can be considered in vitrectomy for simple RD with sufficient removal of subretinal fluid [26] and pars plana vitrectomy with a partial air tamponade [27].

#### 4.1.2. Expansile Gases

Expansile gases such as SF_6_ and C_3_F_8_ are used more commonly for vitrectomy, and both are heavier than air. These gases are also colourless, odourless, and nontoxic, rendering them ideal for use as vitreous substitutes [17,18]. The buoyancy of these gases maintains the position of the retina against retinal pigment epithelium (RPE) [28]. Expansile gases will be spontaneously reabsorbed in 6 to 80 days and replaced with aqueous humour as seen with air, avoiding a secondary surgery for the removal of the gas [29]. SF_6_ expands within 2 days lasting for 1 to 2 weeks in the vitreous cavity while C_3_F_8_ expands within 96 h and can last for 6 to 8 weeks [7,30]. This expansive property of these gases requires patients to avoid high altitudes (including air travel) for 2 weeks and 6 weeks, respectively, following the administration of these gases [31]. Adverse effects of expansile gases include raised intraocular pressure after surgery, cataract formation, foveal sensitivity, and endothelial changes to the cornea [12,32,33]. Temporary vision impairment due to the difference in refractive indexes between the gas and surrounding ocular tissue is also observed [28,32]. When vitrectomy is performed under general anaesthesia, and these gases are used as a tamponade, dinitrogen monoxide (N_2_O) must be avoided. Studies show that due to the strong diffusion tendency of N_2_O, a rapid expansion of expansile gases is caused leading to a fast increase in intraocular pressure, and in some cases, the retinal artery is occluded resulting in a temporary or permanent loss of vision [34]. Expansile gases are suitable short-term vitreous substitutes, but alternatives should be used for long-term substitution.

### 4.2. Liquids

Liquid vitreous substitutes include balanced salt solutions (BSS), perfluorocarbon liquids (PFCLs), semi-fluorinated alkanes, silicone oil, and heavy silicones. They have been studied and used as endotamponades that can respond to head movement without being absorbed and degraded.

#### 4.2.1. Balanced Salt Solutions (BSS)

The physical characteristics of balanced salt solutions (BBS) such as the refractive index, density, and optical transparency are similar to those of the aqueous humour [18]. These solutions are used as irrigating solutions rather than vitreous substitutes and may change once injected due to proteins, metabolites, and cells in the vitreous cavity [35,36]. Balanced salt solutions have a low surface tension leading to no tamponade properties on the retina that are required by an ideal vitreous substitute [12].

#### 4.2.2. Perfluorocarbon Liquids (PFCLs)

Perfluorocarbon liquids (PFCLs) are chemicals in which fluorine atoms replace all the hydrogen atoms [37]. Because of their physical features such as high specific gravity, low surface tension, low viscosity, and optical transparency, PFCLs are an appropriate alternative for vitreoretinal surgery [37,38,39]. However, due to the long-term toxicity, PFCLs are limited to intraoperative use where they flatten the retina temporarily allowing for the repair of retinal detachments [7,38,40]. This toxicity results in mechanical damage to cells, disorganisation of the retina, and ocular inflammation [38,41,42]. The low viscosity of PFCLs allows for injection, removal, and tissue manipulation [38]. PFCLs have a high oxygen solubility and a study with perfluorotributylamine (FTBA) as a vitreous substitute in rabbits showed that FTBA had a neuroprotective effect [43].

#### 4.2.3. Semifluorinated Alkanes (SFAs)

Semifluorinated alkanes (SFAs) are physically and chemically inert, colourless, and immiscible in water. SFAs have a lower specific gravity than PFCLs and this allows them to produce less retinal damage than that observed with PFCLs [44]. SFAs can be used as long-term tamponades as their refractive index is close to that of the vitreous in the human eye [45,46]. Adverse effects linked with the use of SFAs include emulsification and cataract formation [45,47]. The solubility of SFAs in silicone oil has led to their combined use to overcome the emulsification of SFAs alone [48].

#### 4.2.4. Silicone Oil

Silicone oil (SO) is the gold-standard for long-term vitreous substitution [12]. SO is a polymerised liquid siloxane that is hydrophobic. SO polymers are the most used vitreous substitute as they have high surface tension and viscosity, are easy to remove, have low toxicity, and are transparent [7,25,49]. These properties make SO substitutes the ideal long-term vitreous replacement. SOs are used for complicated RD, in uncooperative patients such as children and adults with physical impairment, and when postoperative air travel is planned [12,50]. Viscosities of 1000 to 5000 centistokes of SO are used [49]. SO removal is typically performed between 3 and 6 months after the initial surgery when the retina is deemed to be adequately attached [12,49]. SOs have a tamponade effect on the superior retina while preserving anatomical integrity because of their high surface tension and low specific gravity [51]

Patients need to be monitored closely following SO administration as it has many disadvantages. Its low specific gravity means that it is difficult for SO to tamponade the inferior retina. SO has a refractive index higher than that of ocular tissue and thus optical adjustments need to be made. Other disadvantages include emulsification, increased intraocular pressure, intraocular inflammation, decreased choroidal thickness, oxidative damage causing cataracts, and glaucoma [49,50,52,53]. Complications that may arise following SO administration require the SO to be removed. Removal of SO can also lead to recurrent RD, corneal abnormalities, hypotony, and a loss in the clarity and sharpness of the patient’s vision [52,54].

#### 4.2.5. Heavy Silicone Oil

Heavy silicone oil is created by mixing silicone oil and SFAs forming a homogenous tamponade [12,18]. The combination of the two creates an effective tamponade that is heavier than water and has minimal emulsification in comparison to SFAs alone [47,55]. Heavy silicone oil has been used for complex RD involving inferior proliferative vitreoretinopathy [48,56,57]. Complications associated with heavy silicone oil include cataract formation, emulsification, elevated intraocular pressure, and anterior segment inflammation [58,59].

## 5. Experimental Polymeric Vitreous Substitutes

Hydrogels are three-dimensional (3D) polymers. Without dissolving, they can swell in aqueous solutions, and thermosetting hydrogels are a type of bio-responsive hydrogel that swell to a gel (undergo sol-gel, solution-gel, transitions) in response to a change in temperature [12,60]. Due to their ability to gel at physiological temperatures, thermosetting hydrogels can be developed to have a tamponade effect in the eye [12,61].

Polymeric hydrogels have the potential to be the ideal vitreous substitute as they can be injected as aqueous solutions, after which they form a gel in situ in response to a change in temperature. An in situ gel is a solution that can transition into a semisolid gel in response to a physiological change such as pH and temperature [12]. This ability to form a gel in situ is advantageous as it overcomes the problem of shearing and degradation when a hydrogel is injected through a small gauge needle [20,62]. Some disadvantages that need to be overcome when using polymeric hydrogels include short degradation times [12], retinal toxicity [63,64,65], polymer fragmentation [62,66,67], and an increased time to form a gel within the eye [68].

### 5.1. Natural Polymers (Polysaccharides and Proteins)—Structural Vitreous Substitutes

Natural polymers are proteins (silk, collagen, gelatin, fibrinogen, elastin, keratin, actin, and myosin), polysaccharides (cellulose, amylose, dextran, chitin, and glycosaminoglycans) or polynucleotides. Natural polymers degrade relatively easily, and thus synthetic polymers have been considered a more ideal option for long-term substitutes due to more prolonged degradation [12]. However, natural polysaccharides and proteins can be modified to retard their degradation [69]. These modifications include chemical and morphological modifications such as the introduction of functional groups on reactive groups and the introduction of other polymers to form polymeric complexes [70]. Studies utilising natural polysaccharides and polymers for vitreous substitution were conducted due to the similar molecular structures between these polymers and the natural vitreous. The focus of these studies was to fabricate a vitreous substitute that would mimic the structure of the natural vitreous in its chemical composition. Natural polysaccharides and polymers such as hyaluronic acid and collagen are components of the natural vitreous rendering them one of the first options for investigation for vitreous substitution [66,71].

#### 5.1.1. Hyaluronic Acid

Hyaluronic acid (HA) is a natural polysaccharide that is found in the vitreous of the eye [71]. HA is biocompatible, mucoadhesive, and biodegradable making it ideal for ocular drug delivery [72]. Since the 1970s HA has been studied as a vitreous substitute [73]. Through the extraction and purification of sodium hyaluronate, a highly viscous, clear, and injectable HA solution was derived. This solution was then found to be biocompatible in a clinical trial. However, the study that was conducted could not substantiate its application following vitrectomy surgery.

HA is an ineffective intraocular tamponade due to its short degradation time. Several studies have been conducted to extend the degradation period and make HA a suitable vitreous substitute through the functionalisation of HA via crosslinking and copolymerisation. To extend the degradation period of HA, it was crosslinked with another chemical, adipic dihydrazide (ADH), by two different groups. Su et al. [74] oxidized HA to form oxi-HA after which a clear, colourless, and transparent hydrogel was formed following crosslinking with ADH. This hydrogel had a refractive index similar to that of the natural vitreous, the gel matrix could be maintained over 35 days, and it was found to be non-toxic when evaluated on RPE cells. Preliminary studies of this hydrogel in rabbits showed no significant irregular reactions for 3 weeks. Schramm et al. [75] assembled different hydrogels by crosslinking HA with ADH and by photo-crosslinking HA with N-vinyl-pyrrolidinone and UV-light. Both hydrogels that were assembled were clear, transparent, and found to have refractive indexes similar to that of the natural vitreous. Degradation studies of the hydrogels showed only slight degradation over 1 month. No toxicity was observed in RPE cells for the UV-crosslinked hydrogels; however, the ADH crosslinked hydrogel showed mild toxicity unlike that in the study by Su et al. Schramm et al. concluded that hydrogels based on UV-crosslinked HA may be promising vitreous substitutes.

Healaflow^®^ is a commercially available implant that is used in glaucoma surgery as a space-filler to limit post-operative fibrosis [76]. It is a transparent hydrogel that consists of over 97% water and crosslinked sodium HA, with a refractive index of 1.34. These properties of Healaflow^®^ led researchers to study it as a vitreous substitute following PPV in rabbits. Their findings indicated that the hydrogel maintained its viscous structure for a few weeks following vitrectomy and following long-term follow-up, retinal detachments were found to be self-limiting. These results lead the researchers to believe that Healaflow^®^ may potentially be an effective short-term tamponade but more studies need to be conducted to prolong the structural integrity of the hydrogel [77].

Enzymatic crosslinking of silk fibroin with HA has also recently been done to form a hydrogel for vitreous substitution. Using horseradish peroxidase and H_2_O_2_, crosslinked silk-HA hydrogels were formed. Crosslinking of these two polymers resulted in a hydrogel that overcame the limitations placed on the individual polymers with increased stability of the hydrogel and tunability of the rate of gelation of the hydrogel. The resultant silk-HA hydrogel had a refractive index of 1.336 and light transmission of up to 91% [78] but more research needs to be done to improve degradation and to assess the cytotoxicity of the hydrogels for it to be considered for use as a vitreous substitute.

Vitreous substitutes based on crosslinked thiolated HA have also been studied. The first study, by Schnichels et al. [79], prepared two different hydrogels, soft (1% HA) and strong (2.2% HA), from thiolated HA by the formation of disulphide bridges. The researchers found that the thiol-modified HA had the capability to form stable hydrogels by the natural formation of disulphide bridges without the addition of any chemical crosslinkers. Both hydrogels displayed all characteristics required of an ideal vitreous substitute. In addition to this, both hydrogels also showed superiority to silicone oil in that cataract formation was less frequent with the hydrogels. The soft hydrogel prepared by Schnichels et al. with 1% HA was then further studied by Januschowski et al. [80]. The purpose of the second study was to characterise the biophysical properties (intraocular pressure and retinal integrity) of the prepared substitute. Their results showed no significant increase in intraocular pressure and no significant toxicity which justifies in vivo studies of the hydrogel for biocompatibility.

Other modifications to HA include the introduction of other polymers to form polymeric complexes. Nakagawa et al. [81] used HA and collagen, showing that the concomitant use of collagen and HA is capable of a tamponade effect. Their study showed that the mixture is safe and effective for 3 months in rabbit eyes as a vitreous substitute. They also found that the half-life of collagen was enhanced by HA, but an accumulation of collagen was seen on the retina. The concomitant use of HA with gellan as a vitreous substitute has also been studied. The study found that although a gel formed, its application was limited due to the diminished mechanical properties of the gel. CaCl_2_ was added to form a crosslinked hydrogel which resulted in a substitute that could act as short-term tamponade, circumventing the limiting rheological properties displayed by the previous gel [82].

#### 5.1.2. Collagen and Its Derivatives

In the 1960s, polygeline, a derivative of gelatin which itself is a derivative of hydrolysed collagen, was implanted in rabbit and human eyes. A 3.5% solution initially was opaque but remained clear thereafter for 3 months. However, RD and haemorrhages were seen in the rabbit eyes. The retention rate of polygeline decreased to 22.5% by the tenth day and higher concentrations of polygeline cause white dots and inflammation. Copolymerisation with other polymers caused cloudiness and it was concluded that the inflammation caused by polygeline made it unacceptable as a vitreous substitute [83]. Studies conducted by Pruett et al. [66,67] reported many adverse effects following the administration of collagen as a vitreous substitute. These include inflammation, blurred vision due to opacification of the vitreous, and decreased mechanical properties of the gel due to fragmentation.

As discussed previously, a study by Nakagawa et al. found that the hydrogel copolymer of collagen and HA was capable of tamponade, and the half-life of collagen was enhanced by HA but collagen accumulated on the retina [81]. The use of methylated collagen was then studied to determine its efficacy in overcoming previously found disadvantages with collagen. A solution of Type I/II collagen and acidified anhydrous methanol was produced. This solution had no surface tension preventing a tamponade effect making it unsuitable for retinal detachment [84]. Overall, the inflammation and ocular pain caused by collagen and its derivatives render them unsuitable as vitreous substitutes.

#### 5.1.3. Gellan Gum

Gellan gum is a microbial heteropolysaccharide used in the food industry. Gellan gum can form a gel in situ and is capable of maintaining a gel structure at body temperature [85,86]. Suri and Banerjee [82] studied polymers of gellan gum and hyaluronic acid as discussed previously. The resultant gel was crosslinked with CaCl_2_ which formed a stronger hydrogel that could be a short-term tamponade.

#### 5.1.4. Chitosan

Chitosan is a partially deacetylated derivative of the polysaccharide, chitin, and has been extensively used due to its favourable biological properties [87,88]. An in vivo rabbit eye study with chitosan and HA showed no significant effect on the intraocular tissue and no changes in the intraocular pressure were observed. The intra-vitreous chitosan also had a longer biodegradation time than HA with similar inflammatory effects, making chitosan more suitable than HA [89].

Hydroxypropyl chitosan, a derivative of chitosan, has enhanced solubility, stability, and biocompatibility compared with chitosan [88,90]. Jiang et al. [91] studied an in situ forming hydrogel by crosslinking hydroxypropyl chitosan with alginate dialdehyde. Properties such as the refractive index, light transmittance, and density of the prepared hydrogel were comparable to that of the natural vitreous and biocompatibility studies showed the hydrogel to be nontoxic. In vivo rabbit studies showed that the hydrogel caused no change in intraocular pressure 90 days post-operation. A minor decline in vision was seen along with a decrease in the densities of photoreceptors in the eyes, indicating that further studies of this hydrogel need to be conducted.

### 5.2. Synthetic Polymers—Functional Vitreous Substitutes

Some of the shortcomings of polysaccharides for long-term vitreous substitution have led to more research into the use of synthetic polymeric hydrogels. The extensive studies utilising synthetic polymers for vitreous substitution have centred on reproducing the mechanical and viscoelastic properties of the natural vitreous. The focus of these studies was to design a vitreous substitute that would mimic the function of the natural vitreous. The synthetic polymers discussed below have varying chemical structures and properties that allow for tuning of their functions. Synthetic polymers are synthesised via chemical reactions such as polymerisation that utilise harsh solvents and other polymers that could be incompatible with ocular cells. Therefore, due to the use of polymers that may vary notably in composition from the chemical make-up of the natural vitreous such as polyacrylamide [92] and polyethylene glycol [93], in addition to the chemical processes involved in the synthesis of these polymers, intensive biocompatibility studies are essential for clinical application of these substitutes [63,64].

#### 5.2.1. Poly(1-Vinyl-2-Pyrrolidone)

First studied by Scuderi in 1954, Poly(1-vinyl-2-pyrrolidone) (PVP) was the first synthetic polymer to be studied as a potential vitreous substitute. Variable concentrations of PVP solutions were injected into rabbit eyes and no histological reactions were seen but opacification in the eyes occurred [94].

Researchers subsequently studied a selection of PVP gels to select the most suitable materials that have the potential to be vitreous substitutes through various characterisation studies [95]. The 1-vinyl-2-pyrrolidine monomer was then chosen and polymerised with divinyl glycol as a crosslinker. The transparent hydrogel that formed had a similar viscosity and density to the natural vitreous with a refractive index of 1.339 and a shear storage modulus higher than the shear loss modulus [96]. The hydrogels were then tested in rabbit eyes. Macrophages were present after 4 weeks indicating inflammation, and biodegradation of the hydrogel was also seen [97,98]. PVP hydrogels displayed similar properties to the natural vitreous; however, polymer fragmentation from small-gauge needle injection resulted in a decrease in the mechanical properties of the hydrogel [62].

#### 5.2.2. Hydroxypropyl Methylcellulose and Methylcellulose

One of the most abundant naturally occurring polysaccharides is cellulose and synthetic modifications to cellulose are easily accomplished. These modifications have resulted in polymers with improved properties [99]. Examples of modified cellulose that have been used as potential vitreous substitutes include hydroxypropyl methylcellulose and methylcellulose.

Hydroxypropyl methylcellulose (HPMC) is prepared by treating the alkali cellulose fibres with propylene oxide and methyl chloride [99]. A solution of HPMC was injected in rabbit eyes by Fernandez-Vigo et al. In their study, it was observed that HPMC elimination occurred within 10 weeks after implantation deeming the solution unsuitable for long-term vitreous substitution and the sealing of retina holes in RD [100]. The residence time of HPMC was controlled by varying the molecular weight of HPMC in another study. The results indicated that HPMC with a molecular weight of 120 kDa had a half-life of 38 days. However, HPMC was still unsuitable for long-term vitreous substitution [101].

Heating of cellulose fibres followed by treatment with methyl chloride or methyl iodide forms methylcellulose [99]. Methylcellulose can form reverse thermo-responsive hydrogels that can undergo a sol–gel transition when heated [102]. Katagiri et al. [68] prepared a thermosetting gel with methylcellulose mixed with polyethylene glycol. Various gel concentrations were prepared until a final, optimised preparation, WTG-127, was used for further studies. WTG-127 formed a gel at 36 °C and retained transparency upon gelation. However, the time to form a gel was 50 min which resulted in the drifting of WTG-127 under the retina through the retinal tear. WTG-127 is hydrophobic and could not provide a tamponade effect. Thus, WTG cannot be a suitable long-term vitreous substitute, but research can be done on the application of WTG-127 as a drug delivery medium.

#### 5.2.3. Adcon-L

Adcon-L is a polymer that has been used in neurosurgery, formed with proteoglycan esters in gelatin saturated with water [103]. Adcon-L was studied by De Jong et al. [63] as a vitreous substitute in rabbits. After 1 day, ocular inflammation was observed, lens capsules were thickened after 2 weeks, and after 3 weeks, corneal opacity and additional thickening of the lens capsule were observed. Additionally, after 3 weeks, the hydrogels were no longer visible and retinal toxicity was indicated rendering Adcon-L an unsuitable vitreous substitute.

#### 5.2.4. Pluronic Polyol

An aqueous solution of pluronic polyol F-127 (PF-127) is liquid at low temperatures forming a clear gel at physiological temperature. Davidorf et al. evaluated PF-127 in rabbits as a vitreous substitute. Although it formed a gel in situ, severe retinal toxicity was observed, making PF-127 clinically unsafe to use [64]. Severe retinal toxicity of PF-127 was also observed by Hwang et al. [65].

#### 5.2.5. Poly(N-Isopropylacrylamide)

Strotmann et al. [104] studied a polyelectrolyte hydrogel composed of sodium acryloyldimethyltaurate and acrylic functionalised poly(N-isopropylacrylamide) macromonomer crosslinked with glycerol dimethacrylate. They observed that the crosslinked polyelectrolyte was thermosensitive and enabled the hydrogel to form a gel in situ and the developed hydrogel was also biocompatible. Following the promising results from the 2011 study, Strotmann et al. [105] investigated the stability of the developed polyelectrolyte hydrogel against enzymatic biodegradation and conducted further biocompatibility studies. In this study, an absence of cell proliferation was observed suggesting that the integrity of the retinal barrier is not affected by the hydrogel. These studies, however, did not evaluate the optical and mechanical properties of the hydrogel and further studies would need to be conducted to evaluate the capability of these hydrogels as vitreous substitutes.

#### 5.2.6. Polyacrylamide

**Figure 3:**
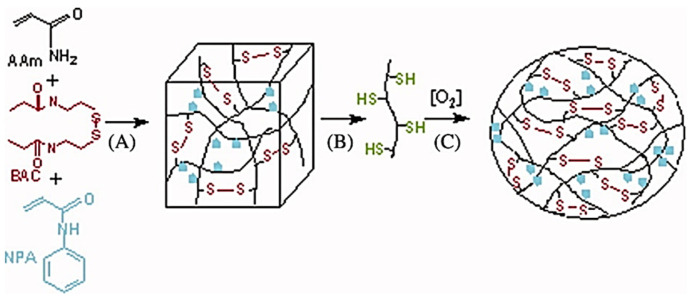
Polymerised acrylamide reversible hydrogel; (**A**) Polymerisation and crosslinking, (**B**) reduction, and (**C**) in situ regelation [20].

Acrylamide is toxic and carcinogenic. However, polymerised acrylamide, poly(acrylamide), is biocompatible, hydrophilic, and viscoelastic, making it an advantageous polymer for vitreous substitution [92]. Reversible hydrogels composed of acrylamide and bis(acryloyl)cystamine (BAC) with disulphide crosslinkers were developed (Figure 3). Reduction of the disulphide bonds to thiol groups resulted in the removal of the toxic monomer. As is needed with current substitutes, following vitrectomy with this hydrogel, a patient would be required to remain face down due to the swelling nature of the hydrogel [106]. After injection into human cadaver eyes as well as porcine eyes ex vivo, studies indicated that the gel elasticity of the developed hydrogel was maintained [15,107]. Foster et al. [107] also confirmed that the developed hydrogels form gels in the eye and osmotic pressure in the vitreous cavity is generated. Further studies showed that the addition of N-phenylacrylamide (NPA) to the previously developed hydrogel increased biocompatibility. Rheological testing of the new hydrogel indicated that the storage and loss moduli matched that of the porcine vitreous [20]. In vivo rabbit studies of the hydrogel indicated no inflammation or toxicity after 1 week [108]. Recently, Davis et al. [21] incorporated acrylic acid into the hydrogel formulation. Various concentrations of this hydrogel were developed and characterised. Results of this study indicated that this new hydrogel could form a gel at lower concentrations while remaining optically clear and biocompatible, thus making it an ideal potential for vitreous substitution.

#### 5.2.7. Methacrylamide

Liang et al. [109] studied and formulated copolymers composed of methacrylamide (MAM), methylacrylic acid, and N’,N’-bis(methylacryloyl-cystamine) (BMAC). Methacrylic acid increases the anionic charge of the copolymer system while also increasing the swell ability. This allowed the researchers to modify the hydrogel to have comparable mechanical properties to the natural vitreous. Liang et al. [109] modified the storage modulus of the hydrogel by changing the copolymer concentration and thiol content. They found that the hydrogels with a lower BMAC concentration were biocompatible, while those with a higher concentration were not. However, the biocompatible hydrogels with a lower BMAC concentration were too soft to be used as vitreous substitutes.

#### 5.2.8. Poly(Glyceryl Methacrylate)

Studies with Poly(glyceryl methacrylate) (PGMA) have been conducted by Daniele et al. [110] and Hogen-Esch et al. [111]. Both studies found that the resultant hydrogel was soft and swelled with analogous optical properties to the natural vitreous. The hydrogel in both studies was also biocompatible; however, the dehydration process in the study by Daniele et al. was found to be too slow and the polymer has been found to fragment upon injection [12,110,111] making PGMA unusable as a polymer for vitreous substitution.

#### 5.2.9. Poly(Methyl 2-Acrylamido-2-Methoxyacetate)

Poly(methyl 2-acrylamido-2-methoxyacetate) (PMAGME) was used to formulate hydrogels that would be stimuli-responsive by Chirila et al. [112]. Various polymers and copolymers of PMAGME were synthesised and tested in rabbits. There was no fragmentation of the polymer when injected; however, the PMAGME hydrogel was found to induce inflammation and scarring, along with being toxic to neural tissue causing the shrinking of nerves in the eye. This study showed that PMAGME is an unsuitable polymer for vitreous substitution.

#### 5.2.10. Polyvinyl Alcohol (PVA)

Polyvinyl alcohol (PVA) has good optical properties, which makes it an ideal polymer choice for a vitreous substitute [18]. PVA was investigated and studied extensively by Yamauchi et al. [113] They first developed γ-irradiated PVA hydrogels that were injected into rabbit eyes and compared to a saline solution. Although the polymer had effective optical properties, inflammation in the rabbit eyes was seen. Yamauchi et al. then formulated a PVA hydrogel mixed with chondroitin sulfate. This hydrogel absorbed more water and displayed a transparency higher than that observed in the previously formulated PVA hydrogel. However, they found that the PVA/chondroitin sulfate hydrogel was less biocompatible than the first hydrogel.

Formulation of PVA hydrogels by gamma-irradiation was also done by Maruoka et al. [114]. In this study, the polymer was autoclaved before gamma-irradiation and injection into the eyes of crab-eating macaques. The purification improved the biocompatibility of the PVA hydrogel. Although an increase in intraocular pressure was seen 1–2 weeks after the operation, normal intraocular pressure and retinal activity were seen after 3 months. Some vacuolizations of the inner retina and low toxicity were observed following the operation, and difficulties were experienced with the injection of the preformed hydrogel.

Methacrylated PVA (PVA-MA) hydrogels were synthesised by Cavalieri et al. [115]. A photo-initiator in the PVA-MA polymer enabled the gel network to be formed when irradiated. Changing the concentration of the photo-initiator as well as changing the irradiation time controlled the degree of crosslinking. Low degrees of crosslinking displayed partial degradation and the storage modulus of the hydrogel was higher than that of the physiological vitreous. Low polymer concentrations could not form a gel and high polymer concentrations formed gels that were much stiffer than the natural vitreous making this hydrogel unsuitable as a substitute.

Leone et al. [116] and Lamponi et al. [117] synthesised PVA hydrogels with a non-toxic crosslinker, trisodium trimetaphosphate (STMP). Leone et al. synthesised PVA/STMP hydrogels of varying concentrations to determine the hydrogel most similar to the natural vitreous. The 1:8 (STMP/PVA) molar ratio hydrogel was recognised as the most suitable following rheological testing and other characterisation tests. This 1:8 ratio hydrogel also showed suitable injectability with no change in its viscoelastic properties. Biocompatibility studies of the STMP/PVA hydrogel were conducted by Lamponi et al. Three ratios of the STMP/PVA hydrogel (1:4, 1:6, and 1:8) were analysed for their effects on various mouse and human cell lines. The 1:8 STMP/PVA hydrogel demonstrated the best biocompatibility to the cell lines used and histopathology examinations of the retina showed no loss of tissue with all retinal layers remaining intact. The hydrogels also demonstrated stability during swelling for 6 months at physiological temperatures indicating their suitability for long-term substitution. The 1:8 STMP/PVA hydrogel also showed no change in its biocompatibility following injection through a 19-gauge needle. The results from the two studies conducted show promising results for the PVA/STMP hydrogels as long-term vitreous substitutes.

#### 5.2.11. Polyethylene Glycol (PEG)

Polyethylene glycol (PEG) is a polymer that is formed by radical polymerisation, and it is hydrophilic. It is biocompatible, resistant to protein absorption, and can form hydrogels making them a good polymer choice for vitreous substitution [118,119]. In the last 10 years, researchers have extensively studied PEG-based hydrogels as vitreous substitutes. Pritchard et al. [120] studied a 5% PEG solution in a rabbit-eye model. Seven days following PPV with a 20-gauge needle showed no change in the translucence, however, complications such as transient hypotony, retinal detachment, and cataracts were observed. The viscosity of the substitute was also observed to have reduced over the follow-up period. All of these complications were likely due to not using any crosslinker with the polymer.

Annaka et al. [121] functionalised PEG with hydrophobic ends to develop a thermo-responsive in situ forming a hydrogel. The hydrogel was clear, transparent, and biologically and chemically inert. The hydrogel was shown to be rigid enough to act as a tamponade; it was nonabsorbable, nonbiodegradable, and injectable through a small-gauge needle. In vivo testing of the hydrogel showed no retinal toxicity and normal intraocular pressure. High polymer concentrations in the hydrogel resulted in moduli higher than the natural vitreous; thus, more research is required for this hydrogel to be a suitable vitreous substitute. A different approach was studied by Tao et al. [122] They developed a system composed of two PEG-based components. Each PEG-based component had a different functionalised end group (thiol and active vinyl). The two components were then mixed and injected into rabbit eyes where they then crosslink in situ. The hydrogel composed of the two components has similar mechanical and optical properties to the human vitreous. Furthermore, the hydrogel was transparent and stable during the 9 month study period. No significant adverse effects were observed during the in vivo rabbit study, demonstrating that chemically crosslinked PEG hydrogels may be suitable long-term vitreous substitutes.

Chang et al. [123] recognised drawbacks in the two-component hydrogel developed by Tao et al. and noted that PEG with the active vinyl end group, PEG methacrylate (α-PEG-MA), can form a gel via free radical crosslinking. This observation resulted in the development of an in situ forming hydrogel based on α-PEG-MA. The hydrogel had a gelation time of 28 min and the stability at body temperature, gel swelling, and gelation kinetics were regarded as suitable for a vitreous substitute. Preliminary in vivo rabbit studies indicated that the hydrogel remained transparent at an acceptable inflammation level. More studies need to be done to fully evaluate the potential of this hydrogel as a vitreous substitute.

To overcome the swelling of hydrogels induced by degradation and high osmotic pressure, Hayashi et al. [93] designed a hydrogel with low polymeric concentrations. The designed hydrogel, Oligo-Tetra-PEG, was developed in a two-step process. The first step forms two Oligo-PEG clusters, Tetra-PEG-SH and Tetra-PEG-MA, by stopping the crosslinking process before the gelation point. The second step involved the mixing of the two clusters to form the hydrogel. By varying the initial polymer concentrations, the researchers managed to control the gelation and mechanical properties of the hydrogel. The osmotic pressure of the hydrogel was lower than eye pressure and it was compatible with small gauge needle surgery. The hydrogel showed biocompatibility before and after degradation and was able to function as a vitreous substitute, treating RD, for over a year in rabbit eyes with no adverse effects seen. The results of this study make this Oligo-Tetra-PEG hydrogel a potential long-term vitreous substitute.

Biodegradation in vivo due to hydrolases [98] resulted in Liu et al. [61] designing a tri-component hydrogel (EPC) that can facilitate vitreous regeneration during the biodegradation of the hydrogel. The EPC hydrogel was formed by linking PEG, poly(propylene glycol) (PPG), and poly(ε-caprolactone) (PCL) with urethane bonds. By changing the polymer concentrations, the researchers were able to tune the gelation temperature of the EPC hydrogel to form a gel at physiological temperatures. The EPC hydrogel was able to form a gel in situ following vitrectomy with a small gauge needle and the hydrogel achieved a surface tension high enough to form across retinal tears. The hydrogel was biocompatible long-term, remained optically clear, caused no intraocular pressure changes or inflammation, and restored normal retinal architecture in RD models. Three months post vitrectomy, total degradation of the hydrogels was observed; however, a vitreous-like body formed in response to this degradation. The vitreous-like body displayed biophysical properties similar to the natural vitreous [61]. This study indicates that EPC hydrogel can function as a vitreous substitute and also enable the regeneration of a vitreous-like body.

During vitrectomy, as the natural vitreous is removed, the oxygen balance in the eye is disrupted, causing oxidative stress resulting in cataract formation. Tram et al. [22] addressed this as a design standard for a vitreous substitute. They designed a hydrogel loaded with an antioxidant to function as a substitute. The hydrogel was prepared by free radical polymerisation of PEG-MA and PEG-diacrylate (PEG-DA). The resultant hydrogels showed comparable optical and mechanical properties to the natural vitreous; it was injectable and showed biocompatibility with human ocular cells. Vitamin C, an antioxidant, was added to the hydrogel to restore oxygen balance to the eye. It was seen that the hydrogel with added Vitamin C had a synergetic effect in protecting retinal and lens cells from reactive oxygen species, indicating that these hydrogels had the potential to act as vitreous substitutes that can prevent the formation of post-vitrectomy cataracts. This is the first reported hydrogel mimicking the chemical function of the vitreous along with optical and mechanical functions.

#### 5.2.12. Other Synthetic Polymers

Other synthetic polymers have also been used to develop vitreous substitutes. Böhm et al. [124] designed hydrogels based on a mixture of polymeric β-cyclodextrin (β-CD) and poly{(2-acrylamido-2-methyl-1-propanesulfonic acid sodium salt)-co-[6-(acrylamido)-N-adamantylhexaneamide]}. The two components of the mixture are cytotoxic but the mixture of the two as a hydrogel was biocompatible. This hydrogel, though biocompatible, is not suitable as a vitreous substitute as the administration of the components is done individually and can lead to cytotoxic effects.

To circumvent the biodegradation of PEG, Chang et al. [125] designed zwitterionic hydrogel that forms in situ. Poly(MPDSA-co-AC) was designed as a copolymer composed of sulfobetaine methacrylamide and acryloyl cystamine. In vitro studies indicated that the hydrogel had suitable gelation properties, viscoelastic properties, and light transmittance for use as a vitreous substitute. In vivo studies showed that the hydrogel remained transparent and displayed no inflammation after 1 month. More studies need to be conducted to assess the viability of this hydrogel as a vitreous substitute.

A zwitterionic monomer unit, carboxybetaine acrylamide (CBAA) was used by Wang et al. [126]. They synthesised a supramolecular copolymer hydrogel composed of poly(N-acryloyl glycinamide-co-carboxybetaine acrylamide). The synthesised hydrogel could hold a water content of greater than 98% and it was stable during swelling by tuning the monomer ratios. The refractive index, modulus, and light transmittance were similar to the physiological vitreous. In vivo rabbit testing indicated that the hydrogel functioned as an ideal vitreous substitute without any adverse effects. This novel approach for a vitreous substitute can be a potential substitute but more studies are required.

Uesugi et al. [127] evaluated a gel composed of a self-assembling peptide, PanaceaGel SPG-127. The physical properties of the gel were comparable to those of the natural vitreous, the gel was biocompatible, and no fragmentation following injection through a 27-gauge needle was observed. Three months after administration, the gel remained transparent, and no complications were seen. The researchers could not verify the tamponade effect of the hydrogel and additional studies need to be conducted before this hydrogel can be a potential vitreous substitute [127].

Xue et al. [128] developed a more optically transparent hydrogel based on Poly[(R)-3-hydroxybutyrate-(R)-3-hydroxyhexanoate] (PHBHx). The physicochemical properties of this polymer along with its ideal machinability were the reason for this choice. The PHBHx hydrogel demonstrated high light transmittance, good sol-gel transition, and rheological properties as a vitreous substitute. Xue et al. [128] showed that increasing the concentration of PHBHx resulted in hydrogel cloudiness. In vivo rabbit studies showed that the hydrogel could maintain transparency when implanted and displayed preservation of the retina and negligible inflammation of ocular tissue over 6 months [128]. This study can be a new direction for research on vitreous substitutes.

### 5.3. Composite Polymers

Hydrogels composed of synthetic polymers alone may not provide a biomimetic effect. Thus, researchers have studied composite hydrogels composed of a combination of synthetic and natural polymers. Ideally, these composite hydrogels would hold the biomimetic properties seen in hydrogels composed of natural polymers and the long-term viability properties observed with synthetic hydrogels.

Lin et al. synthesised a copolymer of HA with Pluronic^®^ F-127. It is an in situ vitreous substitute with ideal chemical, rheological, and optical properties. Biodegradation studies of this substitute indicated that the hydrogel maintained 60% of its mass after 7 days and cytotoxicity studies with RPE cells indicated that the hydrogel did not have any cytotoxic or adverse effects [129].

Three studies conducted since 2016 have evaluated a hydrogel composed of gellan and poly(methacrylamide-co-methacrylate) that forms in situ. Santhanam et al. [130] considered 17 possible hydrogels with varying concentrations of the two polymers to determine how each polymer affects the properties of the final hydrogel. The developed hydrogels had optical and physical properties comparable to that of the natural vitreous with a sol–gel transition temperature range from 35.5 to 43 °C. Degradation studies showed that the hydrogels remained swollen for 4 weeks in vitro and biocompatibility studies with RPE cells showed no toxicity. The hydrogel was found to allow for the optimising of swelling, mechanical properties, and transition temperature to obtain hydrogels similar to the vitreous. Eleven of these hydrogels were then further studied by Santhanam et al. [131] and the two formulations that were most similar to the vitreous were studied further. The concentrations of thiolated gellan and poly(methacrylamide-co-methacrylate) in each of the two formulations were 0.9 g.L^−1^ and 12 g.L^−1^, and 1.5 g.L^−1^ and, 10 g.L^−1^, respectively. Both hydrogels were compared to silicone oil and were found to be clinically acceptable as vitreous replacements. The hydrogels were also biocompatible in rabbits and retained optical clarity and physiological intraocular pressure. The researchers concluded that further long-term in vivo studies of both hydrogels should be conducted [131]. Recently, Laradji et al. [132] further studied the two optimised hydrogels and found them both to closely mimic the natural vitreous. Toxicity studies with various cell lines show biocompatibility, and preclinical studies 4 months after pars plana vitrectomy on rabbits showed no signs of inflammation. The lens was clear apart from partial opacities at the site of surgery. None of the rabbits developed cataracts in the four-month post-operative period. Further evaluation of the hydrogels is ongoing, but it promises to be a superior alternative to current substitutes.

Morozova et al. [133] synthesised a vitreous substitute composed of the methacrylamide (MAM), methylacrylic acid, and N’,N’-bis(methylacryloyl-cystamine) (BMAC); however, unlike Liang et al. [109] they also crosslinked gellan to the copolymer system. They proposed that the addition of anionic charges and a structural component that is fibrous would improve the structural integrity and the density of the vitreous substitute. The synthesised hydrogels swelled and were reinforced by the gellan fibres. Characterisation studies showed that the formulated hydrogel with crosslinked gellan had similar water content, transport properties, and responsiveness to light to the natural vitreous. These results render this substitute a possible ideal long-term vitreous substitute.

## 6. Foldable Capsular Vitreous Body

**Figure 4:**
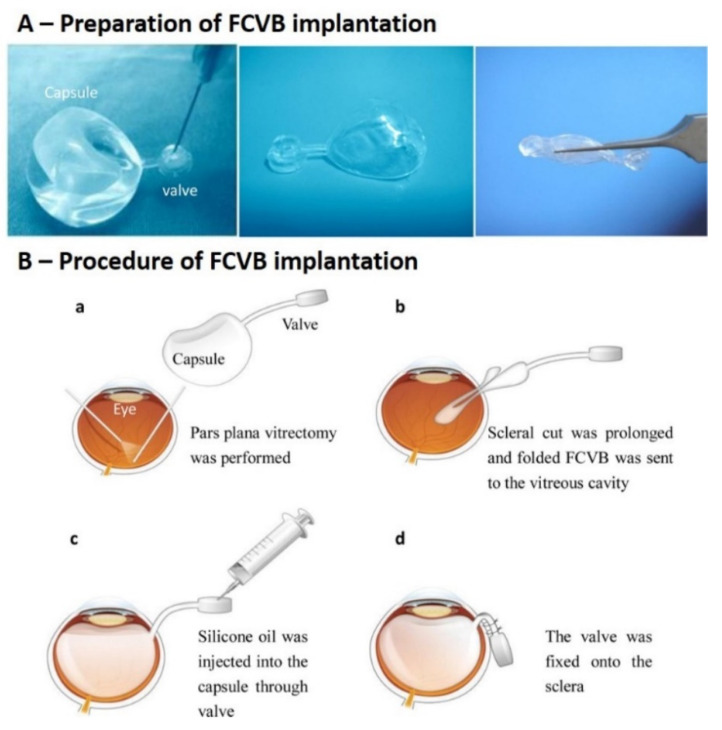
(**A**) Preparation of the FCVB. The airtightness of the FCVB capsule was checked and the capsule was then three-folded like a spindle. (**B**) Procedure for the implantation surgery. (**a**) PPV (**b**) Placement of the FCVB in the vitreous cavity. (**c**) Injection of SO into the capsule (**d**) Fixing of the valve onto the sclera [142].

An alternative to in situ hydrogels has been studied and assessed by a group of researchers. They developed a foldable capsular vitreous body (FCVB) that can be implanted in the ocular cavity and filled. The capsule of the FCVB, with a thickness of 0.01 mm, was composed of a liquid silicone rubber that was vulcanised once the FCVB mold was dipped in it. The liquid silicone rubber material was a crosslinked mixture of polyvinylsiloxane and polyhydrosiloxane [134]. The developed FCVB demonstrated good optical, mechanical, and biocompatible properties while effectively mimicking the physiological and structural functions of the natural vitreous [135,136,137]. The FCVB can be filled with solutions or hydrogels to regulate intraocular pressure, function as a tamponade for the retina, and act as a vitreous substitute. It has been filled with SO, PVA, and PEG (Figure 4) [138,139,140]. Twelve-month and three-year follow-up studies of silicone oil (SO) filled FCVB show promising results. Retinal reattachment was seen at the three-year follow-up. Although some fluctuations in visual acuity were observed, no SO leakage or emulsification was seen, and no glaucoma or other complications occurred [141,142]. The FCVB has also been studied as a drug delivery system [143,144,145,146]. All the FCVB studies indicate an ideal vitreous substitute, and any subsequent studies may result in a new therapeutic strategy for the treatment of RD.

## 7. Future Perspectives on the Design of an Ideal Vitreous Substitute

### 7.1. Drug Delivery

Drug delivery to the retina is a challenge due to the position of the retina in the eye along with the blood–retinal barrier. Intravitreal delivery of drugs for retinal diseases is a direct drug delivery route, however, it still carries the risk of endophthalmitis, vitreous haemorrhage, and retinal detachment [147]. The quick drug elimination from the vitreous requires frequent injections and this would decrease patient compliance [148]. Polymeric nanoparticles retain drugs at the site of absorption for extended periods thus increasing the bioavailability of ocular drugs [149]. Drug delivery via drug encapsulation in nanoparticles is a potentially sustainable long-term delivery route for retinal disease treatment. However, the uncontrolled burst release of drugs from encapsulation [150] and the frequent need for intravitreal injections [151] are disadvantages to the use of nanoparticles alone.

Using a vitreous substitute as a reservoir during drug delivery is a novel delivery system that should be further studied. Such a system can be used long-term and thus increase patient compliance while decreasing adverse effects such as intraocular inflammation, endophthalmitis, and intraocular pressure elevation seen in repeated intravitreal injection administration [152]. The ideal vitreous substitute should be compatible with therapeutic agents to treat common adverse effects seen following vitrectomy such as cataract formation [15,22]. A vitreous substitute with high water content would be able to facilitate drug delivery and drug diffusion through ocular tissues. A vitreous substitute that can act as a drug delivery system will have to be similar to the natural vitreous while also being compatible with therapeutic agents allowing the controlled release of those agents.

### 7.2. Regenerative/Tissue Engineering

Regenerative medicine is a developing field that provides advanced solutions in the repair, regrowth, and replacement of tissues, cells, or organs that are damaged. Several techniques can be utilised such as gene therapy, stem cell and progenitor cell therapy, and tissue engineering [153,154]. Tissue engineering for the regeneration of tissues consists of the development of scaffolds and nano-scaffolds that are biodegradable and biostable for long-term use [155]. Scaffolds for ocular tissue regeneration should have the ability to mimic the extracellular matrix (ECM) and should be porous, biodegradable, and biocompatible to improve the integration of the scaffold into the ocular environment [155,156].

**Figure 5:**
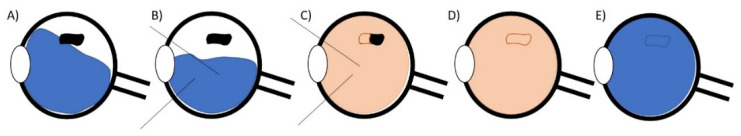
Schematic diagrams illustrating the potential mechanism of vitreous body regeneration following vitreous substitution. (**A**) Retinal detachment with a retinal tear. (**B**) Vitrectomy (surgical removal of the vitreous). (**C**) Intravitreal injection of a gel allowing for chorioretinal adhesion to occur. (**D**) The gel supports the retina as an ideal vitreous substitute. (**E**) The vitreous substitute biodegrades and is replaced by a regenerated vitreous body.

Regeneration of the vitreous humour (Figure 5) would solve many disadvantages that are observed with current artificial vitreous substitutes such as lack of biocompatibility with surrounding tissue, inadequate transport of nutrients, and poor biomechanical properties. The 3D structure of the vitreous is complex and would pose a challenge in the regeneration of the vitreous. Some researchers have investigated vitreal regeneration via hyalocyte control. The researchers controlled hyalocyte proliferation using growth factors and evaluated HA production [157,158]. Regeneration of a vitreous-like body has spontaneously occurred in rabbit eyes following the biodegradation of a PEG-based hydrogel [61] which, together with other research, brings researchers closer to regenerating the natural vitreous.

Challenges with vitreous regeneration are evident in ocular diseases, such as proliferative diabetic retinopathy, that cause damage to the retina and its vasculature [6]. Damage to ocular tissues may affect hyalocyte and collagen regeneration and thus vitreous regeneration. The hyaluronan-collagen network is responsible for the gel nature of the vitreous, which is necessary for its function to maintain the eye’s structure and as a shock absorber. This stiffness of the gel has also been reported to impact stem cell differentiation essential for tissue regeneration [157].

## 8. Conclusions

The vitreous is an essential part of the eye. It has various functions, from filling and shaping the eye to providing stability to the retina. Long-term vitreous substitution is currently only performed using silicone oil while gas substitutes may be used as temporary substitutes. Current long-term vitreous substitutes present disadvantages that have led researchers to study and evaluate other substitutes that may be more ideal. The ideal vitreous substitute must be comparable to the natural vitreous in optical and mechanical properties, demonstrate long-term viability and biocompatibility, and maintain transparency. Recent research on vitreous substitutes has focussed on in situ forming polymeric hydrogels that are injectable via small gauge needles. Hydrogels are an ideal choice for vitreous substitution because they are biocompatible and can act as viscoelastic dampeners like the natural vitreous. Natural polysaccharide-based hydrogels, although structurally similar to the natural vitreous are unable to provide the correct mechanical strength required for vitreous substitution; and synthetic polymer-based hydrogels, while able to function similarly to the natural vitreous, have resulted in retinal toxicity. Manipulation of polymeric hydrogels allows for the tuning of various properties to prevent adverse effects. Substitutes composed of a mixture of natural and synthetic polymers are advantageous as they have the biomimetic and increased degradation profiles required for vitreous substitution. Polymeric hydrogels are a promising future as vitreous substitutes to fulfil patient needs and surgeon requirements.

## Figures and Tables

**Table 1:** Biochemical composition of the vitreous [18].

Biochemical Group	Molecule
Proteins	Albumin
	Transferrin (iron-binding protein)
	Collagen (type II, IV, V, VI, IX, XI)
Glycosaminoglycans	Hyaluronic acid
	Chondroitin sulfate
Metabolites	Ascorbic acid
	Glucose
	Lactic acid
	Amino acids
	Unsaturated fatty acids
	Prostaglandins
	PGE2
	PGF2 alpha
	Prostacyclin
	Thromboxane
Cells	Hyalocytes
	Fibrocytes/fibroblasts
	Macrophages

**Table 2:** Tamponade agents used in the treatment of retinal detachment (Adapted from [7]).

**Silicone Oil (SO) Tamponades**		**Chemical Composition**	**Viscosity (Centistoke)**	**Specific Gravity (g/cm^3^)**	**Interfacial Tension (mN/m)**	**Refractive Index**
Conventional SO						
1000 cSt SO		100% PDMS	1000	0.97	35	1.4
5000 cSt SO		100% PDMS	5000	0.97	35	1.4
Heavy SO						
Oxane HD		88.1% 5700 cSt	3300	1.02	45	1.4
Densiron 68		Oxane/11.9% RMN-3 69.5% 5000 cSt PDMS/30.5% F_6_H_8_	1400	1.06	41	1.4
**Gas Tamponades**	**Chemical Formula and Molecular Weight (g/mol)**	**100% Gas Expansivity**	**100% Maximum gas Expansion**	**Tamponade Duration**	**Isoexpansile Concentration**	**Interfacial Tension (mN/m)**
Air	N/A 28.97	N/A ^†^	N/A ^†^	5–7 days	N/A ^†^	70
Sulfur hexafluoride	SF_6_ 146.06	2×	1–2 days	2 weeks	20%	70
Perfluoro-ethane	C_2_F_6_ 138.01	3×	1–3 days	4–5 weeks	16%	70
Perfluoro-propane	C_3_F_8_ 188.02	4×	3–4 days	8 weeks	14%	70
**Perfluorocarbon Liquids**	**Chemical Formula and Molecular Weight (g/mol)**	**Specific Gravity (g/cm^3^)**	**Viscosity (mPas)**	**Interfacial Tension (mN/m)**	**Refractive Index**	
Perfluoro-*n*-octane	C_8_F_18_ 438.06	1.76	1.20	55.0	1.3	
*Perfluorodecalin*	C_10_F_18_ 462.08	1.33	5.68	57.8	1.3	

^†^ = Not Applicable.

## Data Availability

Not applicable.
